# Co-colonisation with *Aspergillus fumigatus* and *Pseudomonas aeruginosa* is associated with poorer health in cystic fibrosis patients: an Irish registry analysis

**DOI:** 10.1186/s12890-017-0416-4

**Published:** 2017-04-21

**Authors:** Emma Reece, Ricardo Segurado, Abaigeal Jackson, Siobhán McClean, Julie Renwick, Peter Greally

**Affiliations:** 1 Department of Clinical Microbiology, School of Medicine, Trinity College Dublin, Trinity Centre for Health Science, Tallaght Hospital, Dublin 24, Ireland; 2 UCD CSTAR, School of Public Health, Physiotherapy and Sports Science, UCD, Dublin 4, Ireland; 3 Cystic Fibrosis Registry of Ireland, Woodview house, UCD Belfield, Dublin 4, Ireland; 4 Centre of Microbial Host Interactions, Institute of Technology Tallaght, Dublin 24, Ireland; 5 School of Biomolecular and Biomedical Sciences, University College Dublin, Dublin 24, Ireland; 6 Department of Respiratory Medicine, The National Children’s Hospital, Tallaght hospital, Dublin 24, Ireland

**Keywords:** Co-colonisation, Cystic Fibrosis, Aspergillus fumigatus, Pseudomonas aeruginosa, Pulmonary function

## Abstract

**Background:**

Pulmonary infection is the main cause of death in cystic fibrosis (CF). *Aspergillus fumigatus* (*AF*) and *Pseudomonas aeruginosa (PA)* are the most prevalent fungal and bacterial pathogens isolated from the CF airway, respectively. Our aim was to determine the effect of different colonisation profiles of *AF* and *PA* on the clinical status of patients with CF.

**Methods:**

A retrospective analysis of data from the Cystic Fibrosis Registry of Ireland from 2013 was performed to determine the effect of intermittent and persistent colonisation with *AF* or *PA* or co-colonisation with both microorganisms on clinical outcome measures in patients with CF. Key outcomes measured included forced expiratory volume in one second (FEV_1_), number of hospitalisations, respiratory exacerbations and antimicrobials prescribed, and complications of CF, including CF related diabetes (CFRD) and allergic bronchopulmonary aspergillosis (ABPA).

**Results:**

The prevalence of *AF* and *PA* colonisation were 11% (5% persistent, 6% intermittent) and 31% (19% persistent, 12% intermittent) in the Irish CF population, respectively. Co-colonisation with both pathogens was associated with a 13.8% reduction in FEV_1_ (*p* = 0.016), higher levels of exacerbations (*p* = 0.042), hospitalisations (*p* = 0.023) and antimicrobial usage (*p* = 0.014) compared to non-colonised patients and these clinical outcomes were comparable to those persistently colonised with *PA.* Intermittent and persistent *AF* colonisation were not associated with poorer clinical outcomes or ABPA. Patients with persistent *PA* had a higher prevalence of CFRD diagnosis (*p* = 0.012).

**Conclusions:**

CF patients co-colonised with *AF* and *PA* had poor clinical outcomes comparable to patients persistently colonised with *PA*, emphasising the clinical significance of co-colonisation with these microorganisms.

**Electronic supplementary material:**

The online version of this article (doi:10.1186/s12890-017-0416-4) contains supplementary material, which is available to authorized users.

## Background

Cystic fibrosis (CF) is the most common inherited life shortening condition affecting Caucasians. It is estimated that there are over 70,000 people living with CF worldwide [1]. It is a multi-organ disease however up to 95% of morbidity and mortality is caused by airway infections and the associated inflammation [2]. Mutations in the CF transmembrane conductance regulator (CFTR) gene result in dysfunctional or absent CFTR protein which ultimately results in thick airway mucus and impaired mucocilliary clearance. This provides the perfect environment for microorganisms to colonise and persist. The path of disease progression is established early in life with deterioration in lung function beginning as early as age 6 [3]. The recurrent onslaught of airway infection and inflammation results in reduced lung function and eventually respiratory failure.

Airway infection management is the cornerstone of CF care and it is now widely accepted that the CF airways are colonised with a community of microorganisms from an early age [2–4]. Microorganisms previously not considered to be significant in CF microbiology are emerging as having roles in disease progression [4, 5]. The discovery of complex and diverse microbial communities in the airways is forcing a paradigm shift in the treatment of airway infections. Considering this, research into co-infections and polymicrobial infections is imminent. Here we aimed to determine the clinical implications of co-infection with the most common fungal and bacterial pathogen isolated from the CF airways.

*Aspergillus fumigatus* (AF) is the most common fungal pathogen isolated from the CF airways with a reported prevalence between 16 and 58% [6–10]. AF rarely causes invasive infections in CF however it can cause allergic bronchopulmonary aspergillosis (ABPA), a hypersensitivity reaction to *AF* with detrimental consequences on airway inflammation. Generally intermittent or persistent *AF* colonisation is not treated unless ABPA is confirmed. Criteria for diagnosis of ABPA are based on the CF Foundation consensus [10]. At present there is no consensus for the diagnosis or treatment of asymptomatic *AF* colonisation in CF. Recent studies have utilized a combination of AF qPCR, galactomannan, AF specific IgE and IgG to decipher the complexity of *AF* colonisation status in CF patients. Classifying patients into proposed subgroups and delineating those with ABPA from those who are AF sensitised, AF non-sensitised and those with aspergillus bronchitis [11]. Some studies have provided valuable insights into how *AF* may influence the progression of CF lung disease [9, 12, 13] however the true extent of the impact on disease progression is unknown.

*Pseudomonas aeruginosa* (*PA*) is the most common bacterial pathogen in CF [14] and in 2013, the Cystic Fibrosis Foundation in the US reported 48.7% of patients were colonised with *PA* [15]. Early *PA* colonisation of the CF airway is frequently intermittent and eradication is possible using inhaled antibiotics. However re-colonisation can occur and in some cases despite intense efforts to re-eradicate the organism, the colonisation becomes persistent and a decline in lung function ensues [16, 17]. The transition from intermittent to chronic or persistent *PA* colonisation is a seminal clinical event in a CF patient’s life and persistent *PA* colonisation is associated with increased mortality [18, 19].

Despite substantial improvement in median survival, CF remains a life-shortening disease with the mean age of death in Ireland being only 22.8 years [20] and pulmonary insufficiency is the main cause of death. There is a paucity of published data on the prevalence of persistent *AF* colonisation or *AF* and *PA* co-colonisation in CF patients. We aimed to determine the prevalence of persistent and intermittent colonisation with *PA* or *AF* and co-colonisation with both pathogens in the Irish CF population. Furthermore we aimed to determine the association of these different colonisation patterns with the clinical status of patients with CF.

## Methods

### Study population

Data from patients with CF registered in the Cystic Fibrosis Registry of Ireland (CFRI) database in 2013 was used in this study. The number of live patients registered with the CFRI was 1158 on the last day of 2013 (92.5% of the Irish CF population) and data from 749 patients was included in this study (409 patients with incomplete data sets were excluded). A retrospective cohort study was carried out to establish whether colonisation with *AF* and/or *PA* impacts on patient health. The primary clinical outcome measure was forced expiratory volume in one second percent predicted (FEV_1_%) and secondary clinical outcome measures were the number of hospitalisations, respiratory exacerbations (treated with intravenous antibiotics), prescribed antimicrobials and complications of CF, including pancreatic insufficiency resulting in CFRD and ABPA. CFRD diagnosis was defined as being treated with insulin for CF-related diabetes, ABPA diagnosis was reported by the physician.

Sputum (*n* = 1859), throat swabs (*n* = 31), cough swabs (*n* = 52), nasal swab (*n* = 1) and bronchoalveolar lavage (*n* = 10) samples were recorded as positive for *PA* and/or *AF* by the individual hospitals. We employed stringent criteria to classify colonisation status: greater than 2 but less than 4 respiratory samples positive within the year was considered intermittent colonisation and greater than 4 samples positive within the year was considered persistent colonisation, in line with the Leeds criteria [21]. Patients were separated into 6 cohorts depending on their colonisation status: **1)** intermittent *PA* colonisation (*PA*)**, 2)** persistent *PA* colonisation (*PAp*)**, 3)** intermittent *AF* colonisation (*AF*)**, 4)** persistent *AF* colonisation (*AFp*)**, 5)** colonised with both pathogens (*AF + PA*) and **6)** negative for both pathogens (clear). Clinical outcome measures were compared between our six cohorts.

### Statistical analysis

D’Agostino and Pearson omnibus normality test was performed on FEV_1_% predicted data. FEV_1_ data was not normally distributed and outcomes comparisons between groups were made using the Kruskal-Wallis test with Dunn’s multiple comparisons. In order to adjust for the potential confounders: age, gender and CFTR mutation, linear regression was used for FEV_1_% predicted and logistic regression models were used for steroid use, ABPA and CFRD positivity yielding Odds Ratios (ORs) for the “poorer” outcomes.

The number of hospitalisations, respiratory exacerbations and the number of antimicrobials were modelled with a Poisson or Negative Binomial regression. The degree of zero-inflation and over dispersion was assessed by comparing the model Pearson statistic to the sample size for each outcome. In all cases a zero-inflated Negative Binomial model (ZINB) provided the best-fit model yielding Incident Rate Ratios (IRRs). Following this analysis, post hoc tests of all pair-wise comparisons were conducted using a Bonferroni correction for 6 tests (Bonferroni adjusted *p*-value = 6 × *p*-value), and Bonferroni-adjusted 95% confidence intervals (99.583% confidence required). Significance level was assumed at below 0.05. Analyses were conducted in IBM SPSS Statistics version 20 and SAS for Windows version 9.3. Detailed description of data analysis protocols is provided in Additional file 1. All *p* values shown are after adjustment for age, sex and CFTR mutation unless otherwise stated.

## Results and Discussion

### Demographics of the patient population

**Fig. 1:**
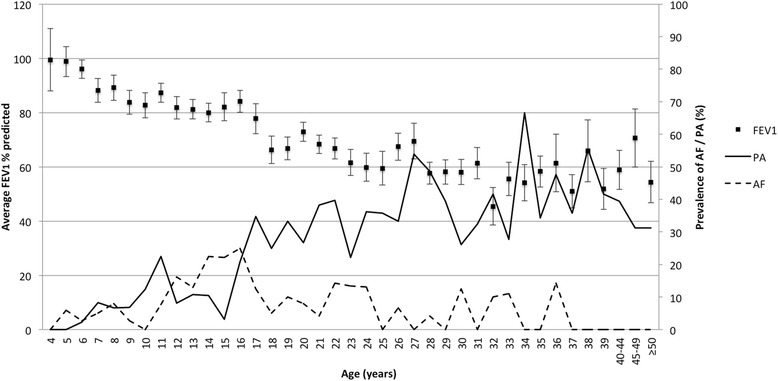
Average FEV_1_ % predicted and prevalence of *PA* and *AF* colonisation in patients stratified by age. Error bars represent standard error of the mean

The male cohort made up the bigger subset of the CF population at 58.6%, reflecting higher mortality rates in females in adolescence and early adulthood [22]. The age distribution in our dataset was 4–69 years and the median age was 18.1 years. The demographic and clinical data of the cohorts are depicted in Table 1. As expected the FEV_1_ percent predicted was lower in older patients reflecting a gradual decline with age (Fig. 1).

**Table 1:** Demographic and clinical data of the study cohorts

		PA	PAp	AF	AFp	AF + PA	Clear
Number (%)		82 (10.9%)	130 (17.4%)	36 (4.8%)	26 (3.5%)	23 (3.1%)	452 (60.3%)
Median Age (range)		26 (7–69)	25 (6–48)	15 (6–36)	15 (5–29)	20 (9–45)	14 (4–56)
CFTR Genotype	phe508del/phe508del	6.8%	10.3%	2.8%	2.9%	1.7%	30.8%
	phe508del/other	3.8%	6%	1.6%	0.4%	1.1%	24.3%
	other	0.5%	0.8%	0.27%	0.13%	0.27%	4%
	unknown	0.13%	0.27%	0.13%	0%	0%	1.2%
Median BMI (range)		21.2^a^ (13.1–34.9)	20 (9–30.2)	19 (14.3–26.8)	18.7 (14.3–26.1)	19 (12.3–25.7)	18.9 (10.4–33.7)

^a^ *indicates missing data*

### Prevalence of AF and PA colonisation in the Irish CF population

The prevalence of *AF* and *PA* co-colonisation was 3.1% in the Irish CF population. The *AF + PA* group included *AFp + PA* (*n* = 4), *AF + PA* (*n* = 5), *AF + PAp* (*n* = 7) and AFp + Pap (*n* = 8) subgroups. No significant differences in FEV_1_, number of hospitalisations, number of respiratory exacerbations and antimicrobial prescribing were noted between these subgroups (Additional file 2: Table S1). *AF* colonisation was most prevalent in pre-adolescents and adolescents (Fig. 1), which contradicts current opinion that *AF* is more commonly an organism encountered in adulthood. To our knowledge this has not been previously reported. The prevalence of *AF* colonisation in the Irish CF population was 11%, of which 5% had persistent and 6% intermittent colonisation (Table 1). Previously *AF* prevalence rates in CF have been reported to be up to 58% using sputum culture [8]. *PA* becomes more prevalent with age and FEV_1_ decline, corroborating previous findings (Fig. 1) [23]. The prevalence of *PA* in the Irish CF population was 31%, of which 19% had persistent and 12% had intermittent colonisation (Table 1). The prevalence of *PA* colonisation in Ireland is lower than the 48.7% prevalence reported in the US [17]. Employing similar criteria for the classification of persistent and intermittent *PA* colonisation as our study, Lee et al., (2003) [21] determined the prevalence of persistent *PA* to be 18% consistent with our data and intermittent colonisation to be 34%, which is higher than our findings. Proesmans et al., (2006) [24] determined the prevalence of persistent and intermittent *PA* colonisation to be 31 and 18% respectively which are higher than our prevalence rates. Many of these studies differ in the criteria employed for categorising colonisation status making comparisons between them difficult. We employed stringent criteria to classify colonisation status in line with the Leed’s criteria [21] as outlined in our methods. The Leed’s criteria have been independently evaluated [24] and are the classification criteria recommended in the European CF Society patient registry guidelines [25]. We did not consider positivity of *AF* and *PA* in one sputum sample in a year as intermittent, as other studies have done [6–8, 26], to rule out inclusion of chance events, false positives or sputum contamination. For these reasons our prevalence for *AF* and *PA* colonisation may be lower than reported elsewhere [6–9, 15, 21, 24, 26]. Consensus criteria for the definition of intermittent and persistent *AF* colonisation, comparable to the Leeds criteria for *PA,* need to be formulated. We acknowledge that a limitation of this study is its cross-sectional design and multi-year analysis of registry data would provide further detail on transition from intermittent to persistent colonisation status and the impact on disease outcome measures.

### Persistent colonisation with PA or co-colonisation with AF was associated with reduced lung function

**Fig. 2:**
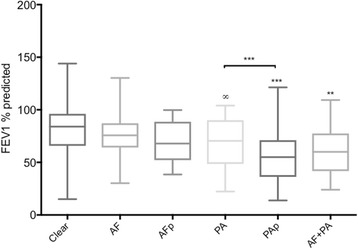
Average FEV_1_ percent predicted in the six colonisation cohorts. Average FEV_1_% predicted for patients with no *PA or AF* colonisation (Clear), intermittent *AF* colonisation (AF), persistent *AF* colonisation (AFp), intermittent *PA* colonisation (PA), persistent *PA* colonisation (PAp) and both *AF* and *PA* co-colonisation (AF + PA). Error bars represent min and max values. Asterisks refer to comparison with Clear, unless over a horizontal bar, ** p ≤ 0.01, *** p ≤ 0.001 (adjusted data). ∞ indicates significance only prior to data adjustment

Patients co-colonised with both *AF* and *PA* had an FEV_1_ 13.8% lower than non-colonised patients (*p* = 0.016) (Fig. 2) and co-colonised patients had comparable reductions in lung function to patients persistently colonised with *PA*. Consistent with our data, Amin et al.*,* (2010) [9] reported that persistent infection with both *AF* and *PA* was associated with lower lung function. The transition of *PA* colonisation from intermittent to persistent is a disease milestone that cannot be reversed and is associated with worse prognosis [18, 27]. We have shown that patients co-colonised with *PA* and *AF* had similar reductions in lung function as patients with persistent *PA*. This is the first time this has been reported. In a recent longitudinal study the sputum microbiology and clinical outcomes of 770 adolescents with CF were recorded and *AF* was the only species that was associated with an increased risk for infection with *PA* [28]. This highlights the importance of monitoring the co-colonisation status of CF patients. Perhaps *AF* colonisation establishes a milieu that enables *PA* to become persistent and attention to *AF* during this phase may prevent progression to persistent *PA*. Further data is required to explore this hypothesis.

Patients with persistent *PA* colonisation had an FEV_1_ 11.9% lower than patients who were intermittently colonised with *PA* (unadjusted *p* = 0.0074)**,** which remained significant following adjustment for confounding factors (*p* < 0.001) (Fig. 2). Previously intermittent *PA* colonisation has been associated with lower FEV_1_ [21] (when adjusted for age and gender only). Following data adjustment for confounding factors (age, gender and CFTR mutation) the FEV_1_ of patients intermittently colonised with *PA* were not significantly different from the FEV_1_ of non-colonised patients. Persistent *PA* colonisation negatively impacted on lung function in agreement with others [21, 23]. Early onset *PA* colonisation can be eradicated in over 90% of colonised patients by antibiotic therapy [29], therefore efforts should be targeted towards early detection of *PA* colonisation and preventing establishment of persistent colonisation. Interestingly 2 studies have now shown that polymerase chain reaction methods can detect PA 4.5 months [30] to 8 months [31] in advance of current culture-based techniques used commonly in diagnostic laboratories. This provides evidence to support the use of molecular-based methods for superior detection of PA*.*

Patients with intermittent and persistent *AF* colonisation had 4 and 11% lower FEV_1_, respectively, than non-colonised patients however these were not statistically significant. A previous study showed patients with *AF* sensitisation or persistent carriage had 16.5% lower FEV_1_ than the control group [32]. Amin et al., (2010) [9] showed that patients persistently colonised with *AF* had a 3.6% decrease in FEV_1_ compared to patients who were clear of *AF,* however when the data was adjusted for baseline pulmonary function, this decrease was not significant. In agreement with our data, other studies suggest no correlation between *AF* colonisation and a decline in pulmonary function [33, 34].

### Persistent colonisation with PA or co-colonisation with both PA and AF was associated with more frequent hospitalisations, more respiratory exacerbations and higher use of antimicrobials

For the first time we have shown that being culture positive for both *AF* and *PA* is associated with similar levels of hospital admissions, exacerbations and antimicrobial prescribing as being persistently colonised with *PA* (Table 2)*.* Patients co-colonised with both *AF* and *PA* had a 165% increase in hospital admissions per person (*p* = 0.023), a 112% increase in the number of respiratory exacerbations per person (*p* = 0.042) and 48% increase in antimicrobial prescribing (*p* = 0.009) when compared to patients who were clear of both pathogens (Table 2)*.* Co-colonised patients had a 64% increase in respiratory exacerbations compared to patients intermittently colonised with *PA* (*p* = 0.015) (Table 2).

**Table 2:** Incident Rate Ratio (IRR) of the incidence of hospitalisations, respiratory exacerbations and prescribed antimicrobials between cohorts

	Hospitalisations		Respiratory Exacerbations		Antimicrobials	
Comparison	IRR (95% CI)	*p* value	IRR (95% CI)	*p* value	IRR (95% CI)	*p*-value
PA vs Clear	1.00 (0.44, 2.30)	1	0.76 (0.37, 1.55)	1	1.19 (0.93, 1.51)	0.259
PAp vs Clear	1.89 (1.11, 3.22)	***0.004***	1.91 (1.19, 3.05)	***<0.001***	1.55 (1.29, 1.86)	***<0.001***
AF vs Clear	1.16 (0.54, 2.48)	1	0.95 (0.45, 1.98)	1	1.63 (1.19, 2.21)	***<0.001***
AFp vs Clear	1.97 (0.75, 5.20)	0.271	1.59 (0.74, 3.44)	0.494	1.9 (1.38, 2.62)	***<0.001***
AF + PA vs Clear	2.65 (1.01, 6.97)	***0.023***	2.12 (0.95, 4.72)	***0.042***	1.48 (1.04, 2.11)	***0.009***
PA vs PAp	0.53 (0.23, 1.23)	0.189	0.4 (0.19, 0.81)	***0.001***	0.77 (0.60, 0.98)	***0.014***
AF vs Afp	0.59 (0.21, 1.67)	0.872	0.59 (0.24, 1.47)	0.775	0.86 (0.56, 1.30)	1
AF + PA vs PA	0.38 (0.12, 1.21)	0.099	0.36 (0.13, 0.95)	***0.015***	0.8 (0.54, 1.19)	0.668
AF + PA vs PAp	1.40 (0.53, 3.73)	1	1.11 (0.51, 2.43)	1	0.96 (0.66, 1.38)	1
AF + PA vs AF	0.45 (0.15, 1.33)	0.209	0.47 (0.17, 1.31)	0.205	1.09 (0.69, 1.74)	1
AF + PA vs AFp	1.30 (0.38, 4.44)	1	0.79 (0.28, 2.21)	1	0.78 (0.49, 1.25)	0.8

Italicized bold text highlights significance of <0.05.

Patients persistently colonised with *PA* had an 89% increase in the number of hospital admissions per person within the year (*p* = 0.004), a 91% increase in respiratory exacerbations (*p* < 0.001) and a 55% increase in antimicrobial prescribing (*p* < 0.001) compared to non-colonised patients (Table 2)*.* In comparison to intermittently colonised patients, those persistently colonised with *PA* had a 60% increase in the number of exacerbations (*p* = 0.001) and 23% increase in antibiotic prescribing (*p* = 0.014) (Table 2). There was no increase in the number of hospitalisations, respiratory exacerbations or antimicrobial prescribing in patients who were intermittently colonised with *PA* compared to non-colonised patients.

Patients intermittently or persistently colonised with *AF* showed no increase in the number of hospitalisations or respiratory exacerbations per person compared to non-colonised patients. However intermittently and persistently colonised patients had a 63% and 90% increase in antimicrobial prescribing compared to non-colonised patients (*p* = <0.001 and *p* = 0.001 respectively) (Table 2)*.* The prevalence of *Aspergillus* sp was previously shown to be higher in CF patients receiving prophylactic antibiotic therapy [33] and we also observed an association between *AF* colonisation and antimicrobial prescribing. We speculate that this finding may reflect antimicrobial suppression of bacteria facilitating fungal growth. It is also possible that *AF* colonisation establishes a milieu suitable for bacterial colonisation, which would impact on antimicrobial prescribing. No differences were observed between the cohorts regarding inhaled steroid use, steroids taken daily and steroids taken alternative days (Additional file 3: Figure S1).

### Patients colonised with PA had a higher prevalence of CFRD

A total of 14.7% of patients with CF had diabetes requiring insulin in 2013. Patients persistently colonised with *PA* had a higher prevalence of CFRD diagnosis than non-colonised patients (*p* = 0.012) (Additional file 4: Table S2). A direct link between insulin deficiency and clinical decline has been shown and patients with CFRD were more likely to be persistently colonised with *Pseudomonas sp.* [35].

### AF colonisation was not associated with prevalence of ABPA

The overall prevalence of ABPA was 5.9%. Patients co-colonised with *AF* and *PA* had the highest prevalence of ABPA (17.4%) but this was not significantly higher than the other groups (Additional file 4: Table S2). Interestingly, patients intermittently or persistently colonised with *AF* showed no increased prevalence of ABPA. It is not currently understood why some *AF* colonised patients are vulnerable to developing ABPA and others are not. A number of single nucleotide polymorphisms (SNPs) have been linked to susceptibility to ABPA. SNPs in the IL-4 receptor alpha chain (IL-4Rα) gene [36], the mannose-binding lectin (MBL) 2 gene [37, 38], the toll-like receptor 9 (TLR9) gene [39] and the pulmonary surfactant protein (SPA-2) gene [40] have been identified as genetic risk factors for the development of ABPA. In particular, SNPs in the promoter region of the IL-10 gene were found to be associated with *Aspergillus* colonisation and ABPA in patients with CF. Further studies in the CF setting could yield interesting insights into the susceptibility of CF patients to *Aspergillus* colonisation and development of allergic disease. Culture of *AF* from respiratory secretions of CF is only a secondary criterion for the diagnosis of ABPA, due to the large number of asymptomatically AF colonised CF patients. A retrospective study on 236 patients with CF found that 25% of patients had positive *AF* culture results but of these only 15 patients (6.5% of the total population) were ABPA positive [41]. In parallel with our findings, positive culture results for *Aspergillus* did not seem to represent a specific risk factor for ABPA [40, 42].

The CF airways are colonised with a diverse community of microorganisms from an early age [2–4]. Interactions between the varieties of dominant and low abundance microorganisms in these communities are likely to impact patient health. The most commonly isolated bacteria and fungi from CF samples are PA and AF, respectively and we have shown that co-colonisation with these two microorganisms is associated with poor clinical outcomes. Previous studies have shown that PA and AF are capable of interacting and competing, which may have implications on CF airway disease. Pyocyanin and 1-hydroxy-phenazine produced by PA both have anti-AF activity [43] and PA has been shown to inhibit AF biofilm formation by direct cell contact and by excreted molecules [44, 45]. More recently the Pf4 bacteriophage produced by PA was shown to reduce both biofilm formation and pre-formed biofilms of AF and this effect was linked to inhibition of AF metabolism via Pf4 iron sequestration [46]. AF has been shown to enhance the production of *PA* elastase when in co-culture and these co-culture supernatants were more damaging to lung epithelial cell lines than PA supernatants alone [47]*.* We hypothesize that these PA-AF interactions may play a role in the damaging pathology associated with CF airway disease and may explain why patients who are co-colonised with PA and AF have a poorer prognosis than those who are clear of both pathogens. Further studies on the interaction of these two microorganisms are warranted to expand our understanding of how co-colonisation may impact the CF airways.

## Conclusions

Persistent *PA* colonisation was associated with more respiratory exacerbations, more hospitalisations and lower lung function than non-colonised patients. For the first time we have shown that co-colonisation with *AF* and *PA* resulted in comparable levels of hospitalisations, respiratory exacerbations and lung function reduction as persistent *PA* colonisation*.* Patients with persistent *PA* had a higher prevalence of CFRD diagnosis. Intermittent and persistent *AF* colonisation was not associated with poorer clinical outcomes or ABPA. This is a cross-sectional registry analysis therefore a prospective longitudinal study is warranted to confirm these findings.

## Additional files

Additional file 1: Supplementary Methods. Further details on the statistics performed on this data (DOCX 140 kb)

Additional file 2: Table S1. Comparison of disease outcome measures between the co-colonised sub-groups (PNG 74 kb)

Additional file 3: Figure S1. Percentage of patients prescribed steroids in each cohort. Percentage of patients prescribed inhaled steroids, oral steroids every day and oral steroids every alternative day for patients within the 6 cohorts (PNG 109 kb)

Additional file 4: Table S2. Odds ratios are presented to determine the odds of a particular colonisation status being linked to development of CFRD and/ or ABPA. (PNG 75 kb)

## Abbreviations

ABPA: Allergic Bronchopulmonary Aspergillosis
AF: Aspergillus fumigatus
AF + PA: AF and PA co-colonised
AFp: Persistent AF
CF: Cystic Fibrosis
CFRD: CF related diabetes
CFRI: CF registry of Ireland
CFTR: CF transmembrane conductance regulator
FEV_1_: Forced Expiratory Volume in 1 second
IRR: Incident Rate Ratios
OR: Odds Ratio
PA: Pseudomonas aeruginosa
PAp: Persistent PA
qPCR: quantitative Polymerase Chain Reaction
SNP: Single nucleotide polymorphism
ZINB: Zero-inflated Negative Binomial
