# Supplementary material for: Co-colonisation with Aspergillus fumigatus and Pseudomonas aeruginosa is associated with poorer health in cystic fibrosis patients: an Irish registry analysis
Source: BMC Pulm Med. 2017 Apr 21;17:70. doi: 10.1186/s12890-017-0416-4 (PMC5401475; doi:10.1186/s12890-017-0416-4)
Supplement: Supplementary file 1 — Supplementary Methods. Further details on the statistics performed on this data (DOCX 140 kb) [file 12890_2017_416_MOESM1_ESM.docx]

**Supplementary Methods**

***Data Analysis Protocol***

In order to adjust for potential confounders: age, gender, and CFTR mutation, regression models were used. Based on the nature of the outcome and inspection of their histograms, the form of the model was decided.

FEV_1_ were approximately Normally distributed, and so linear regression models were used. The five binary categorical outcomes: Inhaled steroids, Daily steroids, steroids every second day, ABPA, and CFRD were modelled using logistic regression which yielded Odds Ratios (ORs) of the “poorer” outcomes (“Yes” for these five variables) for each predictor (PA or AF) group, relative to the reference group (set as “Clear”).

The remaining variables: number of hospitalizations, number of respiratory exacerbations, number of antibiotics/antifungals exhibited varying severity of positive skew. These are all inherently count variables, and so could be modelled with a Poisson or Negative Binomial regression. The degree of zero-inflation (excess number of “zero” counts”) and overdispersion (excess spread for a Poisson distribution) was assessed by comparing the model Pearson statistic to the sample size for each outcome. For all models a zero-inflated Negative Binomial model (ZINB) appeared to provide the best model fit. These models yield coefficients which when exponentiated give Incident Rate Ratios (IRRs) – interpretable as the fold-increase in the incidence of the counted event, relative to a reference group (“Clear”). The ZINB model consisted of a mixture of a binomial distribution predicting the occurrence of zero versus not-zero counts, and a negative binomial model predicting the number of non-zero counts, with the predictor groups (PA or AF) and the confounders allowed to have effects on both the number of counts, and the mix of zero versus non-zero counts.

For both ORs and IRRs, the null value is 1 – that is, the particular group has the same odds or incidence as the reference group. A value less than one indicates a lower odds or incidence, and a value greater than 1 indicates a higher odds or incidence.

The coding for Steroids every second day was Yes or No, where the No group included both patients not on steroids, and patients on steroids every day. For the purposes of these adjusted analyses it did not make sense to collapse those two disparate groups, and so the patients on daily steroids were excluded from the analysis of this outcome.

The principal predictors were two sets of mutually exclusive groups, the first based on PA (PA, PAp, AF+PA, and clear) and the second on AF( AF, Afp, AF+PA, clear). The AF+PA and the Clear patient groups were identical between the two analyses.

Results are presented in the form of unadjusted means (for all groups) or Odds Ratios or Incident Rate Ratios (for all except the reference group) as appropriate, alongside adjusted means, ORs or IRRS from regressions including Gender (male/female), Age (in years) and CFTR mutation (homozygous, heterozygous, other). Where possible (linear and logistic regressions) a p-value (unadjusted and adjusted) is presented from an omnibus test for PA or AF groups – testing whether there is any difference between the four predictor groups.

Following this analysis, posthoc tests of all pair-wise comparisons were conducted using a Bonferroni correction for 6 tests (Bonferroni adjusted p-value = 6 x p-value), and Bonferroni-adjusted 95% confidence intervals (99.583% confidence required). Analyses were conducted in IBM SPSS Statistics version 20 and SAS for Windows version 9.3.
