# Supplementary figures and images for: Co-colonisation with Aspergillus fumigatus and Pseudomonas aeruginosa is associated with poorer health in cystic fibrosis patients: an Irish registry analysis
Source: BMC Pulm Med. 2017 Apr 21;17:70. doi: 10.1186/s12890-017-0416-4 (PMC5401475; doi:10.1186/s12890-017-0416-4)

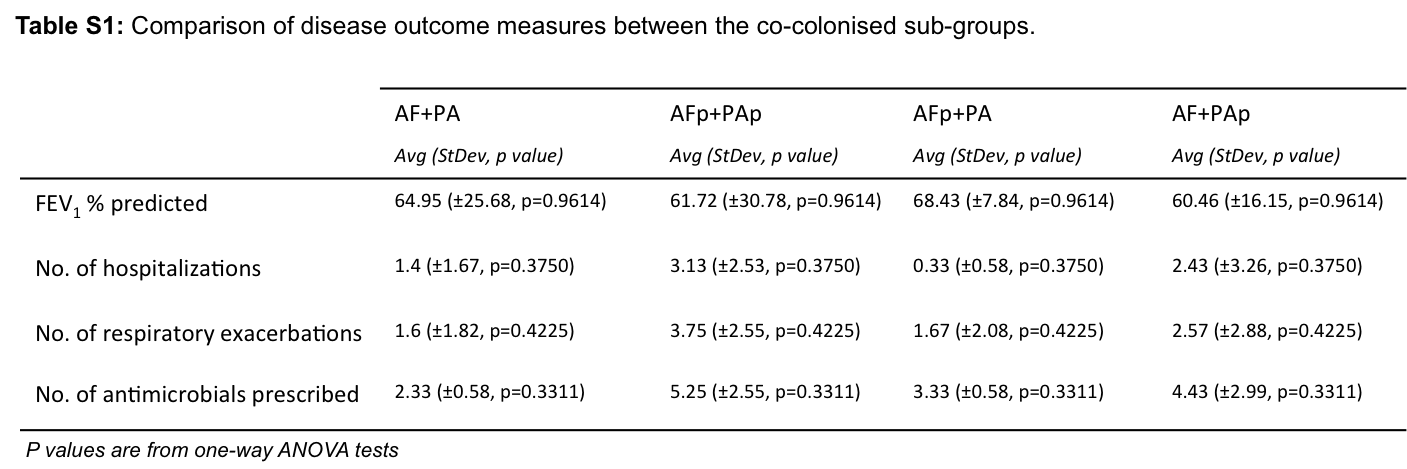

Supplement: Supplementary file 2 — Comparison of disease outcome measures between the co-colonised sub-groups (PNG 74 kb) [file 12890_2017_416_MOESM2_ESM.png]

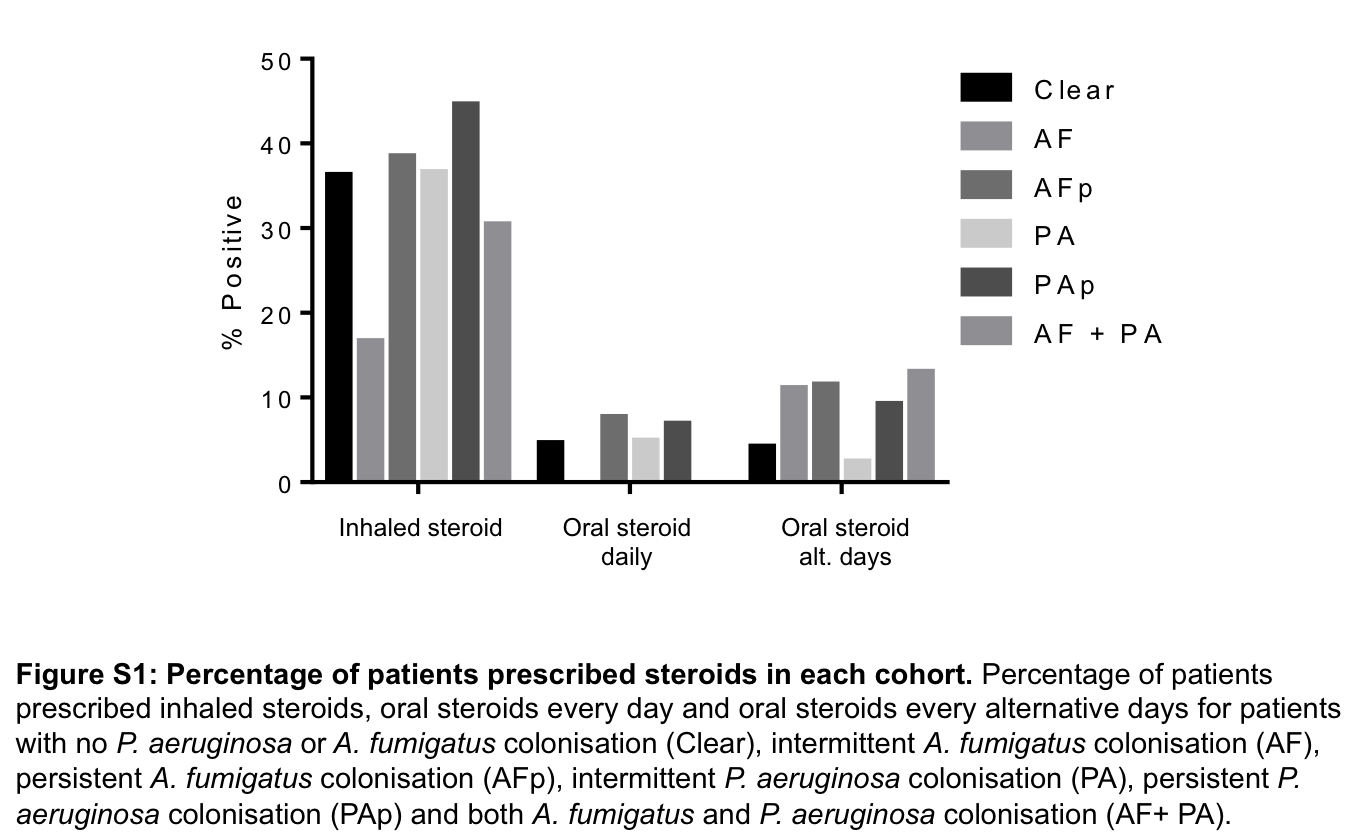

Supplement: Supplementary file 3 — Percentage of patients prescribed steroids in each cohort. Percentage of patients prescribed inhaled steroids, oral steroids every day and oral steroids every alternative day for patients within the 6 cohorts (PNG 109 kb) [file 12890_2017_416_MOESM3_ESM.png]

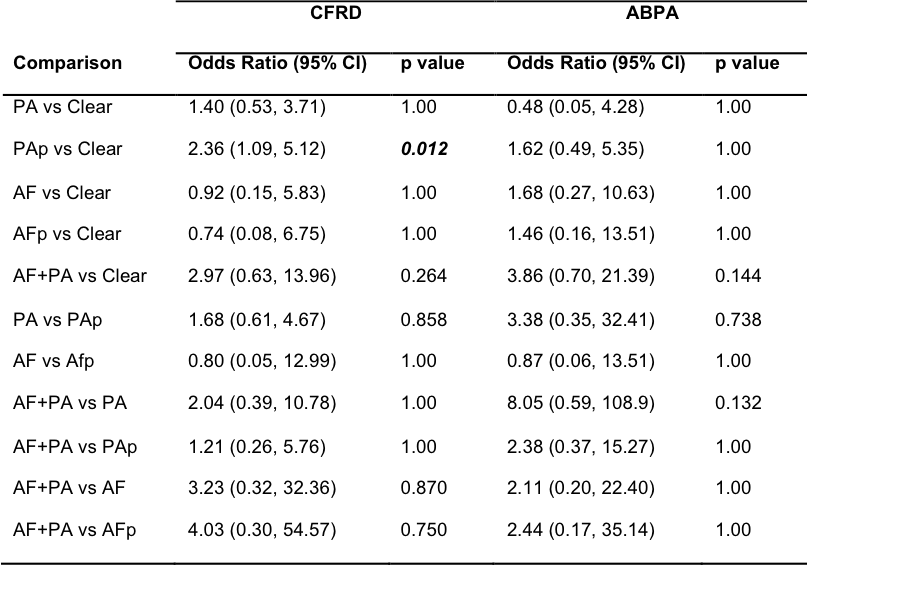

Supplement: Supplementary file 4 — Odds ratios are presented to determine the odds of a particular colonisation status being linked to development of CFRD and/ or ABPA. (PNG 75 kb) [file 12890_2017_416_MOESM4_ESM.png]
